# Many Health Visitors Untrained

**Published:** 1919-01-18

**Authors:** 


### Many Health Visitors Untrained. A Growing Evil.

A Superintendent of a School for Mothers and Infant Welfare Centre, writing on the subject of the inadequate training of tuberculosis and health visitors, to which reference was made in The Hospital of December 21 (Pp. 243-4), says: "I am at times appalled by the ignorance displayed through lack of sufficient training, especially the readiness shown to make a diagnosis." The writer gives an instance of a woman who attended her Centre for kidney trouble, and who had been told by "the lady from the Town Hall" that she was in a consumption, and was to go to the dispensary to be examined. The patient had been given a card for this purpose. It appeared in the course of further conversation that the health visitor had arrived at the conclusion that it was a case of consumption because the woman "coughed," although the patient told the health visitor that she had been treated for kidney trouble.

